# Two potential equilibrium states in long-term soil respiration activity of dry grasslands are maintained by local topographic features

**DOI:** 10.1038/s41598-020-71292-4

**Published:** 2020-08-31

**Authors:** Szilvia Fóti, János Balogh, Bernadett Gecse, Krisztina Pintér, Marianna Papp, Péter Koncz, Levente Kardos, Dávid Mónok, Zoltán Nagy

**Affiliations:** 1 MTA-SZIE Agroecology Research Group, Szent István University, Páter K. u. 1, Gödöllő, 2100 Hungary; 2 Institute of Biological Sciences, Szent István University, Páter K. u. 1, Gödöllő, 2100 Hungary; 3 Duna-Ipoly National Park Directorate, Költő u. 21, Budapest, 1121 Hungary; 4 Department of Soil Science and Water Management, Szent István University, Villányi út 29-43, Budapest, 1118 Hungary

**Keywords:** Ecology, Environmental sciences

## Abstract

Soil respiration of grasslands is spatio-temporally variable reflecting the changing biological activities of the soil. In our study we analysed how the long-term soil respiration activities of dry grasslands would perform in terms of resistance and resilience. We also investigated how terrain features are responsible for response stability. We conducted a 7-year-long spatial study in a Hungarian dry grassland, measuring soil respiration (R_s_), soil temperature (T_s_) and soil water content (SWC) along 15 measuring campaigns in 80 × 60 m grids and soil organic carbon content in 6 of the occasions. Two proxy variables were introduced to grasp the overall R_s_ activity, as well as its temporal stability: average **rankR**_**s**_, the temporal average R_s_ rank of a measuring position from the campaigns revealed the persistent spatial pattern of R_s_, while **rangeR**_**s**_, the range of ranks of the positions from the campaigns described the amplitude of the R_s_ response in time, referring to the response stability in terms of resistance or resilience. We formulated a hypothetic concept of a two-state equilibrium to describe the performance of the long-term R_s_ activity: R_s_ activity with smaller **rangeR**_**s**_, that is both the lower elevation positions with larger **rankR**_**s**_ (“state I”) and the higher elevation positions with smaller **rankR**_**s**_ (“state II”) correspond to an equilibrium state with several terrain attributes being responsible for the equilibrium responses. Majority of the measuring positions was belonging to none of these equilibrium states. These positions showed higher **rangeR**_**s**_ for medium **rankR**_**s**_, suggesting resilience (not resistance) as a major strategy for this ecosystem.

## Introduction

Grasslands exchange large quantities of greenhouse gases between the soil and the atmosphere. Uncertainties related to greenhouse gas flux estimates originate partly from the fact that these fluxes are spatio-temporally highly variable^1–5^.

Seasonal and diurnal fluctuations of these fluxes, e.g., soil respiration (R_s_) and its components are partly temperature (T_s_) driven^6^ but temporal changes in soil moisture (SWC^7^), plant biomass, photosynthetic performance^8^ and litterfall also play a significant role in modifying the overall picture. Also, R_s_ and its main abiotic drivers, T_s_ and SWC, show substantial horizontal heterogeneity at different spatial scales^4,9–13^, which is made even more complex by the interaction of the explanatory variables (e.g., cooling effect of soil moisture^4,11,14^). These point to the relevance of spatial studies with temporal replicates^14^.

Although the actual value and spatial distribution of the pattern-generating factors are responsible for the observed spatial pattern of R_s_, the functioning of ecosystems takes place through dynamically changing, forming and transforming spatial patterns^13,15–19^, which are worth further investigations. Furthermore, the stability of ecosystem functions and the existence of persistent patterns are of high significance as these patterns are sustained by long-term climatic, surface relief and soil conditions and characterise the system’s most general responses, resulting from both resistant and resilient ecosystem responses.

**Figure 1:**
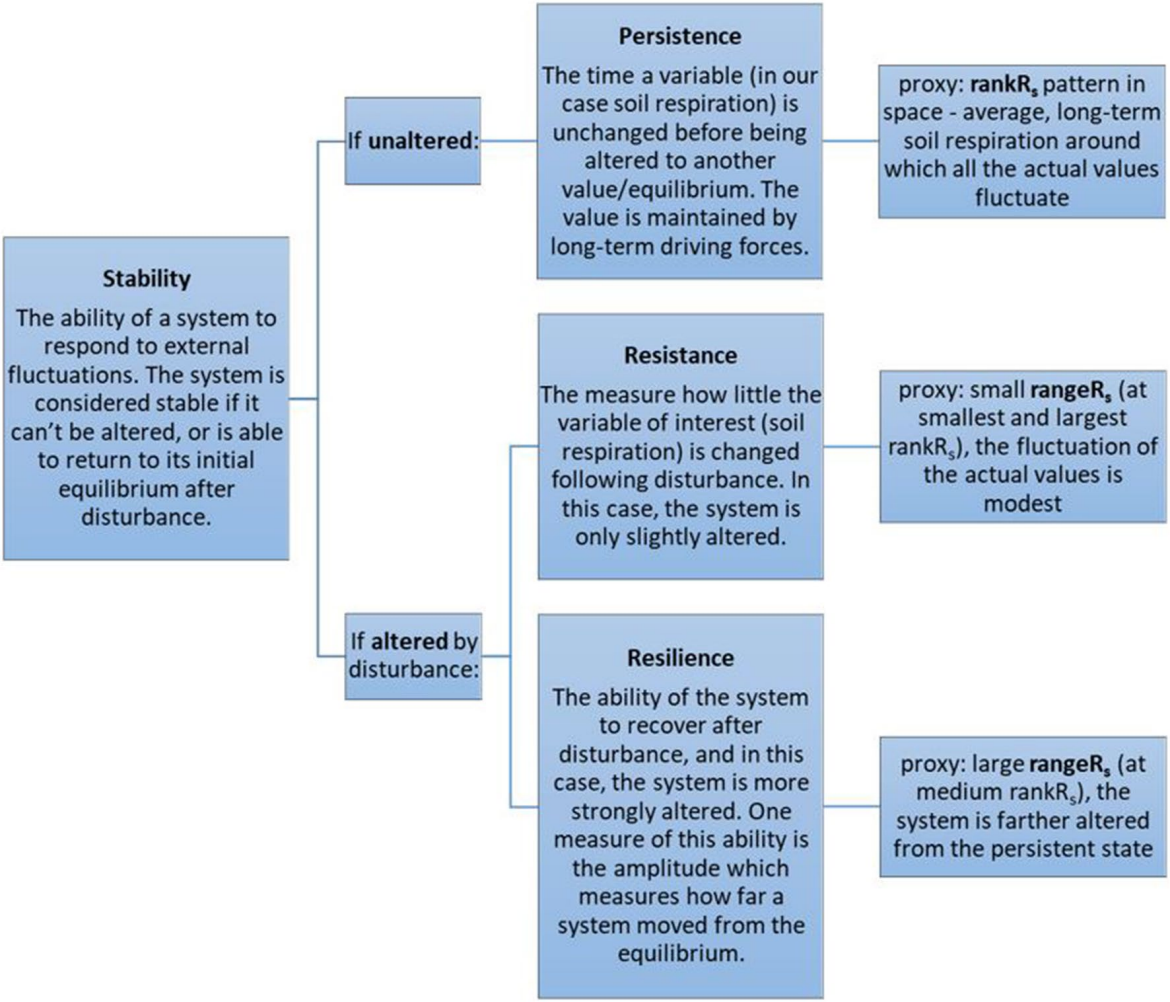
Definitions of the key theoretical concepts and the corresponding proxy variables used in this study.

The key concepts of ecological stability, such as persistence, resistance and resilience are properties hard to quantify and are always context-dependent^20,21^. Following the concepts found in literature^20–23^, we will use those terms as defined in Fig. 1 (see proxy variables in Methods later on). An ecological system is always exposed to a certain level of disturbance, e.g., related to global changes^22,24,25^. In general, an equilibrium system would respond with different amplitude and response time than a perturbed system^20,22^, whether in nutrient cycling or in community dynamics^26^.

Digital elevation models (DEM) are frequently used not only in landform classification^27–31^ or soil mapping^32^ but in ecological studies^18,33^ as well. Detailed spatial analysis of DEM help to capture relevant information about the terrain surface elements, which can have important ecological effects. The slopes and altitudinal differences can be closely related to surface runoff, water accumulation, snow movement or subsurface biophysical processes^19,31,34^, which influence e.g., vegetation patterns or plant species abundance, diversity and distribution^30,33,34^. The aspect as a measure of slope orientation captures different physical and subsequent biological effects related to predominant wind direction and solar radiation (north- and south-facing slopes differ in the duration of shade, snow cover, vegetation period^34^, or a west-facing slope would be warmer than an east-facing slope late in the afternoon), both affecting landscape formation and microclimate characteristics. Other terrain attributes like local mean elevation, standard deviation of elevation within a specific area or topographic position index (TPI) used in our study revealed no co-varying aspects^35^ of the surface.

Compared to the use of DEM in the above-mentioned studies, the effects of the terrain features on the spatial patterns of grassland soil respiration were scarcely studied. Cultivated areas, grazed or restored grassland vegetation types with different aspects and slope positions have mostly been analysed for soil organic carbon, total nitrogen or other nutrient distribution/accumulation/erosion as well as patterns of above and belowground biomass^36–40^. These studies provided evidence for the effects of these terrain features on the differences in the spatial patterns of the soil nutrients or plant biomass, both influencing R_s_ spatio-temporally.

The complexity of the terrain and the study scale have important consequences on the terrain attributes. An ecological phenomenon and an underlying mechanism can have different spatial scales, as in some cases a neighbouring effect can rather act than a single factor at one particular position^34,41^. Matching scales has to be explorative^31^, since it has to be taken into account that some characteristics disappear at broader scales^29^ and that the relative importance of an attribute may change across scales^35^. The picture becomes even more complex if we consider the fact that an ecological function can be influenced by different terrain characteristics^42^ and that an attribute may be involved in different biophysical effects^31^.

In our study we conducted a long-term (7-year-long) spatial investigation in a piece of semi-natural grazed grassland in Hungary. The finely undulating (no more than 1.5 m elevation differences within the study site) surface in our study site is formed through the combined effects of wind, water erosion and drought, resulting in uneven soil nutrient (soil organic carbon, SOC) and water distributions together with different above-ground biomass covers between crests and depressions^18^. We measured R_s_, T_s_ and SWC along 15 measuring campaigns in 80 × 60 m grids and SOC in 6 of these occasions. Some of these datasets have already been used for detailed geostatistical analysis with a different focus (^18^: effects of grassland management on CO_2_ and N_2_O flux spatial patterns). Our current research question formulated on the basis of spatial samplings (15 occasions) was how soil respiration activity of the grasslands would respond to a range of environmental constraints in terms of resistance and resilience in the longterm, and whether the terrain features were responsible for differences in response stability. We hypothesized on the basis of our previous work^18^ that lower micro-elevation levels (surface depressions), rather than the crests, could be responsible for more stable R_s_ activities in general through the effect of more persistent water availability even under drought.

## Results

### Spatial patterns of stability proxies and background variables

**Figure 2:**
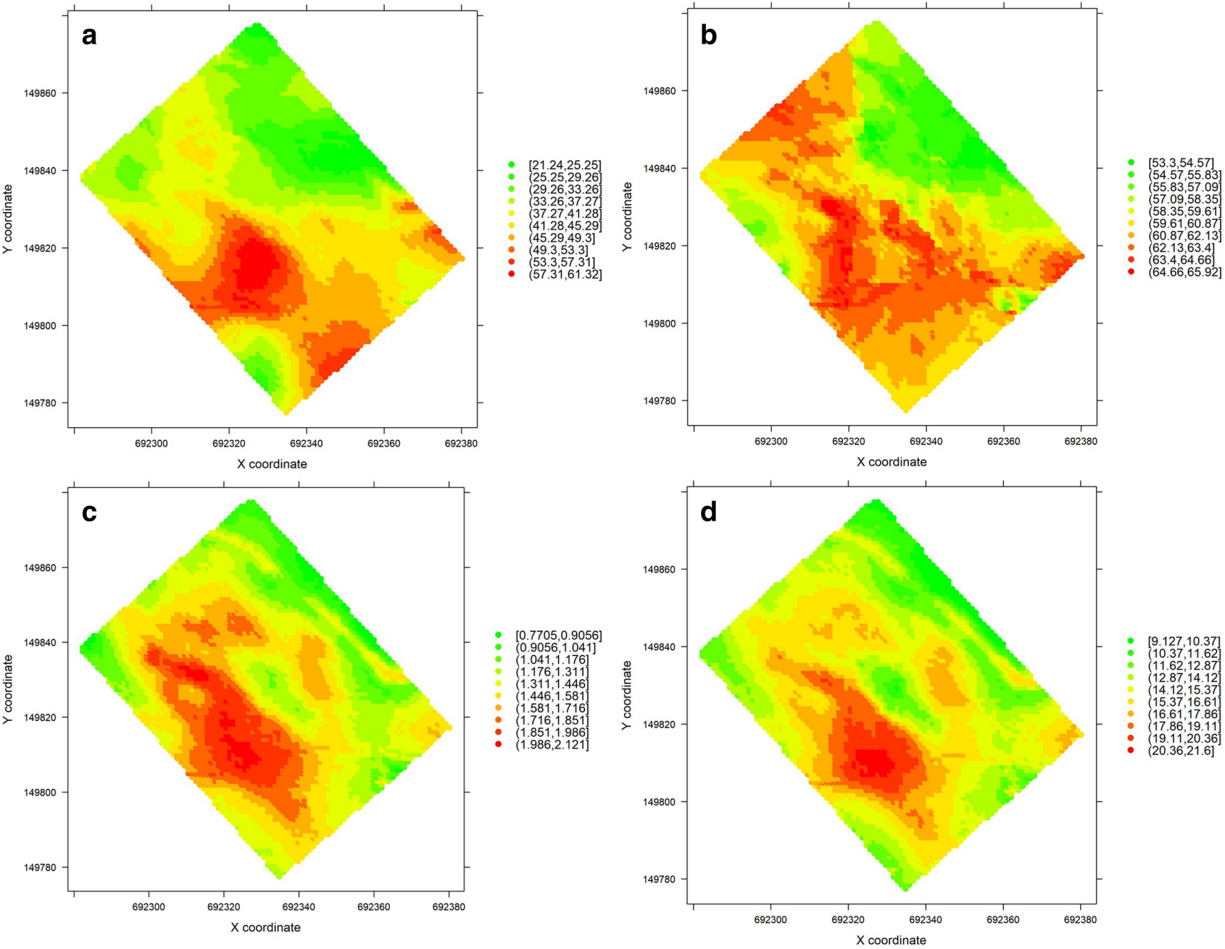
Kriged patterns of stability proxies, rankR_s_ (**a**) and rangeRs (**b**), as well as of background factors, meanSOC (%, **c**) and meanSWC (%, **d**).

Figure 2 a, b show the spatial distribution of our two proxy variables, the average rank of R_s_ per position (**rankR**_**s**_) and of the range of the ranks per position (**rangeR**_**s**_) in kriged maps. The middle to southern areas were found to have the largest, whilst the north-eastern areas the smallest **rankR**_**s**_ values, whereas a slightly different pattern was characteristic for **rangeR**_**s**_ with some additional north-western large values. Similarly, larger average soil organic carbon content (**meanSOC**) and average soil water content (**meanSWC**) (Fig. 2 c, d) were detected at the western-middle-southern regions and smaller at the north-eastern part of the study site.

### Correlations between stability proxies and background variables along DEMs: entire dataset

We investigated the potential direct effects of the different terrain attributes (local mean elevation (*mALT*), standard deviation of elevation (*SD*), topographic position index (*TPI*), slope (*Sl*), Easterness and Northness (*East*, *North*)) on the spatial distributions of our proxy variables by using the terrain attributes originating from differently smoothed DEM rasters. DEM1 was the original, 0.2 m resolution model, while DEMs 2–6 were progressively smoothed by a factor of two resulting in different resolution DEM rasters (DEM2: 0.4 m, DEM3: 0.8 m, DEM4: 1.6 m, DEM5: 3.2 m, DEM6: 6.4 m, respectively), and finally DEM7 met the resolution of the field measuring campaigns (10 m). The terrain attributes were filtered out from the rasters for the 78 measuring positions of the sampling grid.

On the basis of the correlation analysis we found an important difference in terrain attribute features between DEM 5 and 6, especially in *SD*, *Sl*, *North* and *East*. All subsequent results are then based on DEMs 1–5, which were found to be more similar to each other and to the original DEM1. The maps of terrain attributes with the box blur kernel from DEM1-5 can be found in the Supplementary Information (SI) together with the descriptions and calculations. As we couldn’t find any of the blur kernels superior to the other when considering correlations, the results hereafter are only presented for the box blur kernel calculations for simplicity.

**Figure 3:**
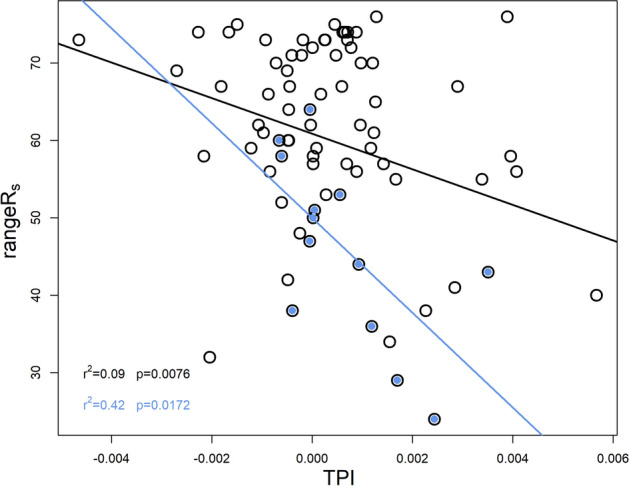
Direct correlation between TPI and stability proxy, rangeR_s_ at less smoothed DEMs, DEM1-2 for datasets A (black symbols and line) and S (blue symbols and line, see the information later on). The correlations were significant at *p* = 0.0076 and *p*  = 0.0172 levels, although they were weak, r^2^ = 0.09, r^2^ = 0.42 for A and S (see the information later on), respectively.

When we considered the entire dataset (named hereafter: “A” dataset), we could only find significant correlation between **rangeR**_**s**_ and *TPI* at less smoothed DEMs but the correlation was very weak (black symbols and line in Fig. 3).

Any other correlation between the proxies and the terrain attributes could only be deduced indirectly from the positive correlations between **rankR**_**s**_ and **meanSOC**, **meanSWC** (cf. Table 1b). These correlations were scale-independent, i.e., we detected them at every DEMs. In general, the larger the soil carbon content and soil moisture at a position (cf. Figure 2c,d, showing quite similar patterns to the proxy patterns in the figure upper row), the larger the **R**_**s**_ activity detected and the opposite was true for lower carbon content and soil moisture positions.

**Table 1:** (a) Statistically significant (*p* < 0.05) linear correlation between terrain attributes, ALT, mALT, TPI, SD, Sl, North, East and background factors, meanSOC, meanSWC for A dataset. (b) Statistically significant (*p* < 0.05) linear correlation between terrain attributes, background factors and stability proxies, rankR_s_, rangeR_s_ in A dataset and in the subgroups (see codes in the text). Regular letters mean scale-independent correlations (valid for DEM1-5), italic underlined letters mean correlations valid for less smooth DEMs (DEM1-2 or 1–3), “pos” and “neg” indicate the sign of the correlation.

(a)	meanSOC	meanSWC	(b)	rankR_s_	rangeR_s_
ALT	A: neg		ALT	M: neg, C1: neg	
mALT	A: neg		mALT	M: neg	
TPI			TPI	*C1*: neg	*A*: neg, *S*: neg
SD	A: neg	A: neg	SD	*C1*: neg, *C5*: pos	
Sl	A: neg	A: neg	Sl	*C1*: neg, *C5*: pos	
North	A: pos		North		*C4*: neg
East	A: pos		East		
			meanSOC	A: pos, C4: pos	
			meanSWC	A: pos, C1: pos, C4: pos, C5: pos	

As we investigated the background of these correlations more thoroughly in dataset A in terrain attributes (cf. the maps in SI, Table 1a), we found that **meanSOC** showed negative correlation with *ALT*, *mALT*, *SD and Sl,* while positive with *North and East* at DEMs 1–5. Similarly, **meanSWC** correlated negatively to *SD*, *Sl*, except for DEM5. Several terrain attributes were then responsible for the **meanSOC** patterns, i.e., higher absolute elevation and neighbouring surface heterogeneity, as well as steeper slope positions facing more South-West could be characterized with lower **meanSOC**. The opposite features were characteristic for higher **meanSOC** level positions on lower elevations with lesser neighbouring heterogeneity and gentle slopes facing mostly North and East. Similarly, **meanSWC** was higher at smaller surface heterogeneity with more gentle slopes, while lower at more heterogeneous surfaces with steeper slopes. **rankR**_**s**_ followed these patterns with higher R_s_ activity in the **middle-southern part of the study area**, while, in contrast, lower R_s_ activities were characteristic at lower **meanSOC** and **meanSWC** at the **north, north-east facing locations** in the study site, mainly on local ridges as found on the basis of the direct *TPI* correlations.

### Correlations between stability proxies and background variables along DEMs: subgroups

We also checked the correlations within different data subgroups corresponding to specific **rankR**_**s**_ or **meanSOC** categories because we hypothesized that these kinds of groupings could enable us to grasp some important characteristics of the stability.

**Subgroups**:

- Subgroups were created on the basis of **rankR**_**s**_ ± SD: **S** (**S**maller than mean-sd), **M** (**M**iddle between mean ± SD), **L** (**L**arger than mean + SD).
- **C1**, **C2**, **C3**, **C4**, **C5** (from the smallest to the largest **meanSOC** quintiles).

Direct correlation between terrain attributes and proxies showed considerable variation depending on the subgroups and variables (Table 1b collects scale-independent correlations, valid at almost each of the DEMs between 1 and 5, or scale-dependent ones, valid only in several of the less smoothed DEMs 1, 2, 3).

It seems that the **meanSOC** pattern related negatively to *ALT*, *mALT* detected in dataset A could have acted as a driver for the negative **rankR**_**s**_ and *ALT*, *mALT* correlations in the groups M and C1. It was in subgroup C1 that *TPI*, *SD* and *Sl* acted negatively on **rankR**_**s**_ as well, most probably more directly through the patterns generated by terrain attributes in **meanSWC**. Further negative correlations were found between **rangeR**_**s**_ and TPI in S data (see also blue symbols and line in Fig. 2), like in dataset A (cf. Fig. 3 black symbols and line), as well as between **rangeR**_**s**_ and North in C4. Accordingly in the long run, local valleys but mostly constant slope positions (with TPI close to zero, cf. blue symbols in Fig. 3) with lower neighbouring surface heterogeneity and gentle slopes with more elevated **meanSOC** and **meanSWC** could be characterized with larger R_s_ activity with higher variability (through the negative *TPI*-**rangeR**_**s**_ correlation) in these subgroups per se, similarly to dataset A. The opposite was likely to be the case for local ridges.

**Figure 4:**
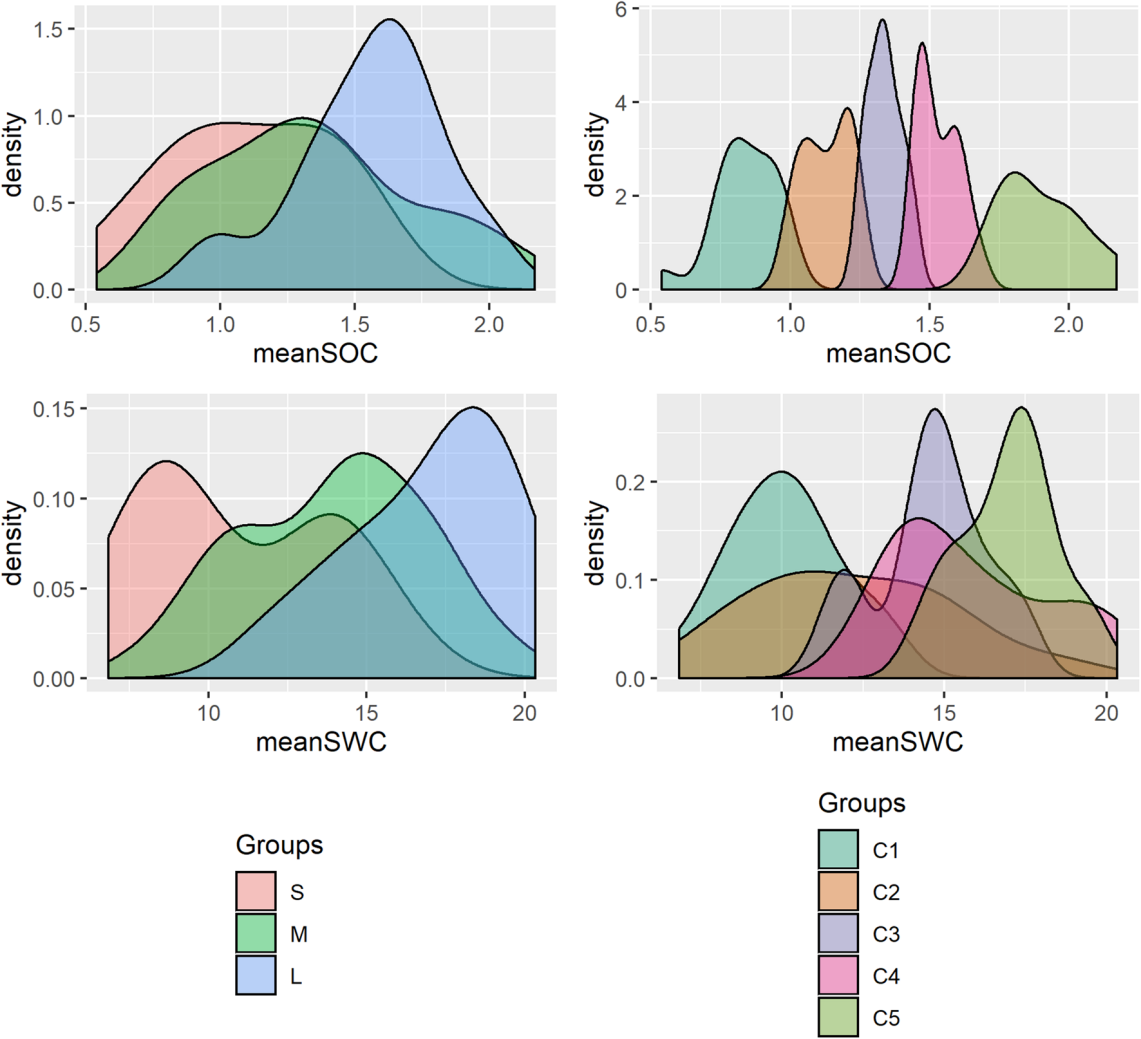
Density plots of meanSOC and meanSWC while grouping the measuring positions according to their rankR_s_ category (S-M-L groups) or meanSOC content (C1–C5 groups).

The subgroups mentioned here were restricted groups of measuring positions, where carbon content in the soil was the lowest of all (C1) or, as in subgroup S, coincided with low **meanSOC** levels (Fig. 4), and low **meanSWC** levels as well.

**Figure 5:**
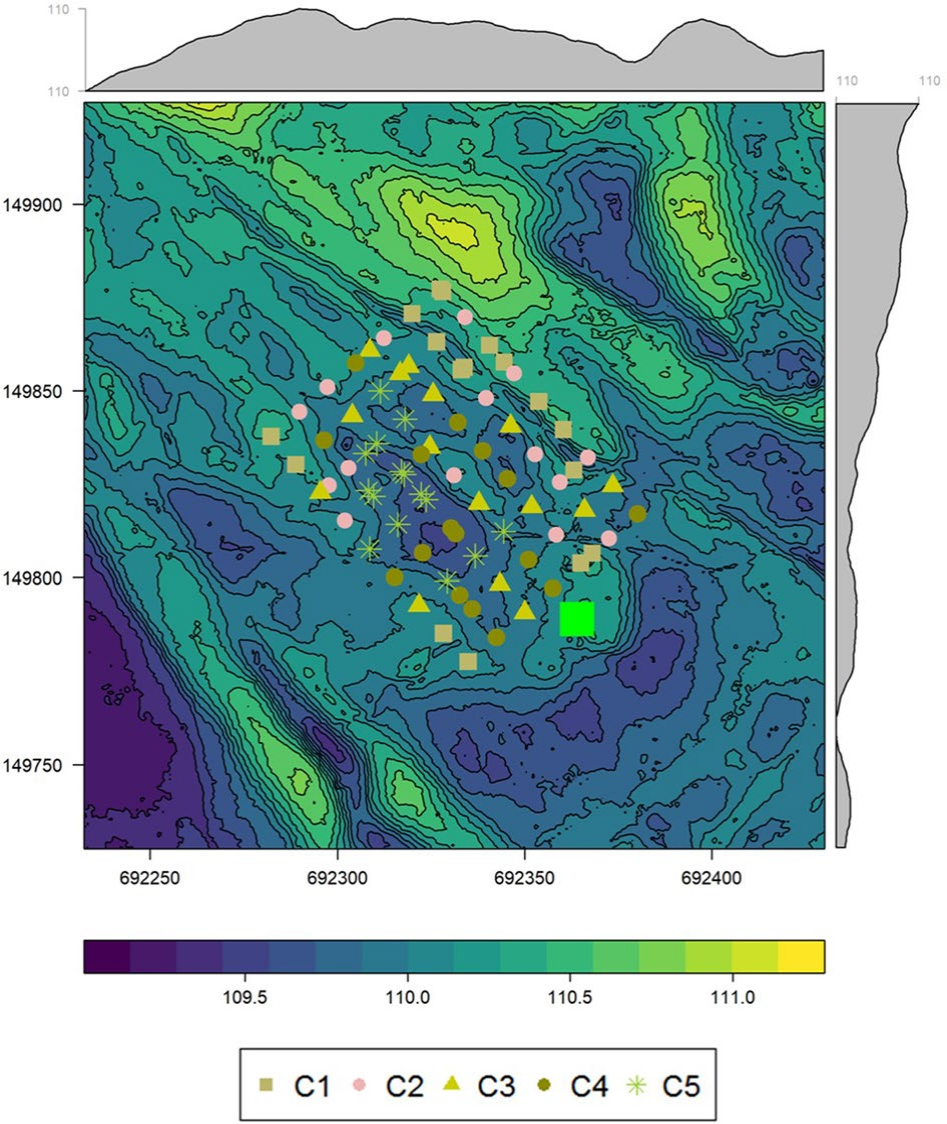
Digital elevation model of the study plot in Bugac, Hungary (coordinates refer to the Uniform National Projection System (m)) with the sampling positions in the 80 × 60 m grid coloured according to their meanSOC from C1 to C5. Green square represents the position of the eddy covariance station. Marginal plots in grey show the mean surface elevation by x and y coordinates. Notice that the largest altitudinal difference was no more than ~ 1.5 m within the study plot.

Furthermore, measuring positions, grouped by either on the basis of **rankR**_**s**_ or on the basis of **meanSOC**, occupied more or less well delimited spatial areas within the sampling grid (Fig. 5, positions coloured according to C1–C5, where e.g., C5 category, indicated with asterisk occupied the lowest altitudinal positions, C4 was found mostly around C5, while C1 category could be found along the edges of the study area on the crests), which would also be characteristically different in respect of the terrain attributes, especially in *SD*, *Sl*, as found by the correlations (cf. Table 1).

**Figure 6:**
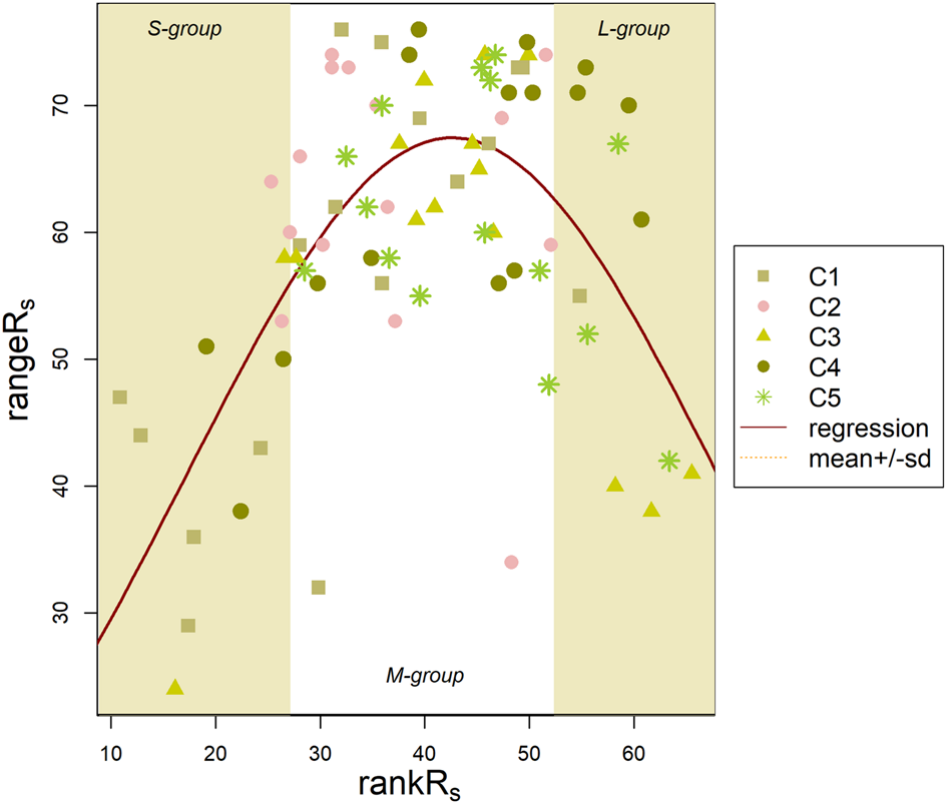
Bell-shaped curve correlation between rankR_s_ and rangeR_s_ visualized together with the two series of subgroups, S-M-L created on the basis of rankR_s_ categories, C1–C5 created on the basis of meanSOC categories. Equation and parameters of the curve are in the main text.

Finally, **rangeR**_**s**_ was fitted to **rankR**_**s**_ using the following equation:

1 $$rangeR_{s} = a \times \frac{1}{{\sigma \sqrt {2\pi } }}e^{{ - \left( {rankR_{s} - \mu } \right)^{2} /2\sigma^{2} }} ,$$

where µ is the mean **rankR**_**s**_ (42.61), σ is the standard deviation of **rankR**_**s**_ (25.38), and *a* (4,291.73) is a model parameter. The correlation, approaching a bell-shaped curve (Fig. 6, both the curve and the model parameters are statistically highly significant, *p* < 0.0001), visualized together with the subgroups showed that both low and high **rankR**_**s**_ could be associated with small **rangeR**_**s**_ with larger stability and a typically resistant response, while middle values of **rankR**_**s**_ corresponded to larger **rangeR**_**s**_ with a more flexible, resilient response of R_s_ activity. Furthermore, it was also showed that **rankR**_**s**_ categories more or less fitted to C1–C5 **meanSOC** categories (cf. square symbols of C1 in S-M-group regions, C2 in M, while asterisks mostly in the upper half of the **rankR**_**s**_ range), giving strong evidence of SOC as a controlling factor in R_s_ stability. The smallest and the largest **rankR**_**s**_ values could correspond to the **largest potential stability** (in terms of resistance) in the activities, **rankR**_**s**_ being either low in general (cf. Fig. 2a north and north-east regions) due to low **meanSOC** and **meanSWC** (Fig. 2c,d) or high (cf. Figure 2a more the middle and southern regions within the study plot) in the opposite cases. On the other hand, medium **rankR**_**s**_ with larger **rangeR**_**s**_ overlapped spatially with C2-C3 and M groups with medium **meanSOC** levels, and these positions showed a more resilient response.

## Discussion

In our analysis we tried to grasp the persistence, as well as resistance/resilience in soil respiration activity, which is regarded as an important ecophysiological functioning of ecosystems. We used two proxies derived from spatial replicate measurements from several years to analyse stability in R_s_ dynamics. R_s_ has an inherent temporal variability due to its environmental control through spatio-temporally varying and co-varying factors^4,10,43^. R_s_ is also exposed to disturbances, which can be either defined as a “sudden shock” ^20^ or as a constant disturbance regime like shifts in climatic conditions due to global changes (e.g., nighttime or daytime warming, change in amount or timing of precipitation etc.^22,24^). Our study site is characteristically exposed to the latter, experiencing frequent droughts and heatwaves in summer demonstrated mostly by NDVI data (Fig. 8a), coinciding with earlier predictions to our East-Central European region^44^.

We revealed the spatial pattern of long-term, persistent R_s_ functioning by mapping the average rank of R_s_ in space from a series of measurements. We could conclude that **rankR**_**s**_ calculated from 15 measurements, irrespective of the actual environmental conditions and plant biomass levels, enabled us to describe the long-term average functioning, while **rangeR**_**s**_ allowed us to have an insight into the dynamics of this process. Spatial patterns have already been found to be similar to a certain degree^15,16,18^ but the pattern similarities are assumed to be more qualitative than quantitative^45^ in the long run. **rankR**_**s**_ showed a general picture of the spatial R_s_ activity but did not provide information about the stability of the process. **rangeR**_**s**_ could be more informative in terms of stability. As it corresponds to the amplitude (if there is an alteration) of R_s_ response in time or to the efficiency component of disturbance response^20^ it can indeed be defined as a measure of the stability in R_s_ response. Smaller **rangeR**_**s**_ means more resistant R_s_ activities against the environmental conditions, mostly drought in our region. Larger **rangeR**_**s**_ could correspond to a more flexible response, reflecting resilient R_s_ activities.

However, our results based on the correlations of our proxies and the terrain attributes as well as on the bell-shaped curve relation between **rankR**_**s**_ and **rangeR**_**s**_ suggest the existence of two types of resistant responses with smaller **rangeR**_**s**_: one at lower elevations with high **meanSOC**, **meanSWC** at gentle slopes with large **rankR**_**s**_, and another at higher elevations, at local ridges with lower levels of **meanSOC** and **meanSWC**, at steeper slopes and increased surface rugosity with lower **rankR**_**s**_. The first type of response coincided with our hypothesis based on our previous work^18^ that R_s_ activity would be more stable at lower elevations where water availability is more constant. However, the other type was a new finding in our system but theoretically equally meaningful because complex systems can have several equilibrium states^19,22^.

In order to characterize the complexity of our system we can rely on the terrain attribute analysis. It was finally restricted to DEMs 1–5 as DEM smoothness was found to have significant effects on terrain attributes^29,46^, slope, aspect, curvature, size of catchment area, which were all found to be affected by DEM resolution but also by the vertical precision. We found significant variability of the terrain attributes within our study area (cf. SI Figs. 1–3) which implies substantial spatial differences both in the environmental conditions and in community structure^30,33,34^. Thus, this spatial variability in the conditions may simultaneously cover the effects the system generally faces and those experienced due to the disturbances. Slope differences were found to be responsible for the water regime in general^31,34^, while aspect for incoming solar radiation and for the surface formation by wind^34^.

Finally, we came to the conclusion that the equilibrium state in our system was dynamic^22^. Todman et al^22^ stated that several “smaller-scale domains of attraction” could exist in complex systems. We concluded that both lower elevation positions with larger **rankR**_**s**_ (“state I”) and higher elevation positions with smaller **rankR**_**s**_ (“state II”) but both characterized with smaller **rangeR**_**s**_ could correspond to an equilibrium state. This theory can also rely on the observations that wet and dry soil moisture patterns with transitory phases between them characteristically occur^45,47^. Wet state is a result of non-local forces, acting on excess water supply, while dry state is locally driven by soil properties, incoming radiation and vegetation^47^. Although semiarid regions are in the dry state most of the time^45^, our C5 positions could correspond to a generally wetter or at least more transitory state which has a generally larger soil moisture variability with some intervention of the above-mentioned non-local forces. C1 and S are typically more locally controlled especially if we consider the importance of *TPI* in these places: local ridges are the most exposed to sunlight and evapotranspiration is strong. These differences in water availability between the two equilibrium states together with **meanSOC** differences are well demonstrated in Fig. 4 showing that generally C1–C5 and S-M-L subgroups experience different levels of **meanSWC** and **meanSOC**.

**Figure 7:**
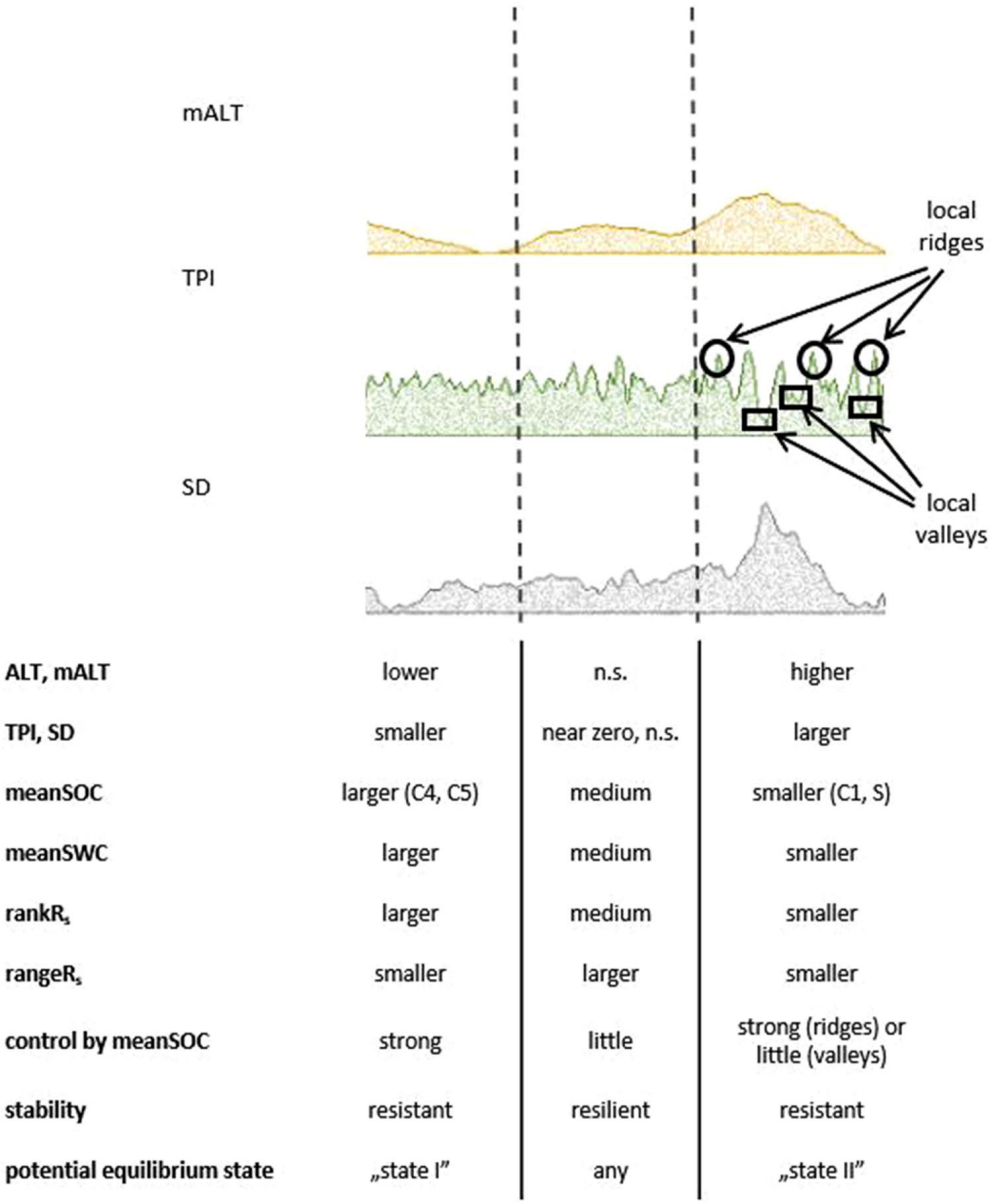
The concept of the two equilibrium states and the potential factors influencing the stability of spatial pattern of R_s_ activity. mALT, TPI and SD “surfaces” presented here were cut as an example from the marginal plots of their similar raster visualizations as DEM in Fig. 5. n.s.: not specified.

On the basis of these observations we attempted to formulate a concept (Fig. 7) concerning the stability of R_s_ activity by trying to grasp the terrain features and background factor characteristics as surrogates^31^.

Several terrain attributes were responsible for the **meanSOC**, **meanSWC** patterns which were found to be the direct spatial drivers of R_s_ activity. Higher absolute elevation (*mALT*) and neighbouring surface heterogeneity (*SD*), as well as steeper slope (*Sl*) positions (Fig. 7 right part: “State II”) could be characterized with lower **meanSOC**, **meanSWC**, mostly on local ridges together with a more resistant response, while the opposite features (Fig. 7 left part, “State I”) were characteristic of higher **meanSOC**, **meanSWC** level positions with lower elevations (*mALT*) with lesser degree of neighbouring heterogeneity (*SD*) and gentle slopes (*Sl*), but also with a more resistant response through a generally larger R_s_ activity. Intermediate levels of the background factors and no specific terrain features characterized the positions (Fig. 7, middle part), where a more resilient R_s_ response was detected.

## Methods

### Site description

The study site is in the Kiskunság National Park, near Bugac (46.69° N, 19.6° E, 114 m a.s.l.), according to a long-term research permission (Grant No: 60960-1-11/2015). The vegetation, which is a semi-arid sandy grassland, is dominated by *Festuca pseudovina* Hack. ex Wiesb., *Carex stenophylla* Wahlbg. and *Cynodon dactylon* L. Pers. The prevailing wind direction is North-West and the surface is slightly undulating. The mean annual precipitation and temperature was 585 mm and 10.6 °C, respectively, for 15 years following the establishment of the eddy-covariance station in 2002. According to the FAO classification^48^ the soil type is Chernozem with a relatively high organic carbon content, the soil texture is a sandy loam with a sand:silt:clay ratio of 81:11:8% in the topsoil layer^49^. The study plot had been under extensive grazing for decades. Grazing intensity was 0.66 ± 0.18 Hungarian Grey cattle animal ha^−1^ year^−1^ for the previous few years. The grazing period usually lasted from May to June and from late August to the end of November. The grassland may potentially turn into a source of carbon in drought years^50^ with the annual Net Ecosystem carbon Exchange (NEE) ranging from -212.6 (sink, 2004) to + 91.2 (source, 2009) g C m^−2^ for the previous 15 years^51,52^.

### Measured variables

The study site was monitored in the vegetation periods between 2012 and 2018 along 15 measuring campaigns (19-10-2012, 08-05-2013, 26-06-2013, 14-10-2013, 07-05-2014, 28-05-2014, 25-09-2014, 09-06-2015, 20-11-2015, 24-10-2016, 02-06-2017, 24-08-2017, 03-11-2017, 17-05-2018, 16-08-2018) similarly to a former study^18^.

Soil respiration (R_s_, µmol CO_2_ m^−2^ s^−1^) was measured by means of closed chamber systems (Licor 6,400, LiCor, Inc. Lincoln, NE, USA and EGM-4 PPSystems, Amesbury, USA) at 78 sampling locations (arranged as a 80 × 60 m grid^18^) in each measurement campaign. Target CO_2_ concentration was set by placing the soil chamber on its side on the ground to monitor the CO_2_ concentration over the surface. Collars were not used with the soil gas exchange chambers (area of 78.54 cm^2^) to minimize disturbance^53,54^ since both measuring systems performed well without collars^55^. Although the sampling positions remained relatively constant for the duration of the experiment, a shift of a few centimetres was applied when selecting the actual patch for each measurement. The standing biomass was removed 1.5 h before starting the soil respiration measurements. To minimize the effects of diurnal temporal patterns the measurements were started at noon and lasted ~ 1.5 h for the grid.

Soil water content (SWC, %) was measured at the same spots as the CO_2_ gas fluxes by time domain reflectometry (ML2, Delta-T Devices Co., Cambridge, UK; FieldScout TDR300 Soil Moisture Meter, Spectrum Technologies, IL-USA) in the top 0–5-cm layer of the soil. The measurements were performed usually after the R_s_ measurements in all positions in one run. Soil temperature (T_s_, °C) was determined at a depth of 5 cm by a digital soil thermometer near the R_s_ chambers parallel with the R_s_ measurements. The soil organic carbon content (SOC, %) of the bulked soil samples from the upper 10 cm was determined by sulfochromic oxidation/loss on ignition.

### Environmental conditions during the study period

Meteorological data were available from the eddy covariance system functioning at Bugac continuously from 2002. The yearly average air temperature, sum of precipitation and NEE data for the study period are shown in Table 2.

**Table 2:** Meteorological conditions and sink-source activity of the grassland during the study years.

	Yearly average air temperature (°C)	Yearly precipitation sum (mm)	NEE (g C m^−2^ year^−1^)
2012	10.7	431	+ 37.9
2013	10.8	590	− 63.5
2014	11.4	813	− 38.4
2015	11.2	523	− 79.8
2016	10.6	584	− 79.5
2017	10.7	654	− 49.8
2018	11.7	578	− 39.1

Annual precipitation sum was lower by 27% in the driest (2012) and higher by 39% in the wettest (2014) year of the study period than the fifteen-year average (585 mm). This variability in water availability resulted in a source activity of + 37.9 g C m^−2^ year^−1^ in 2012, while in a general sink activity between −38.4 and −79.8 g C m^−2^ year^−1^ for the period 2013–2018.

**Figure 8:**
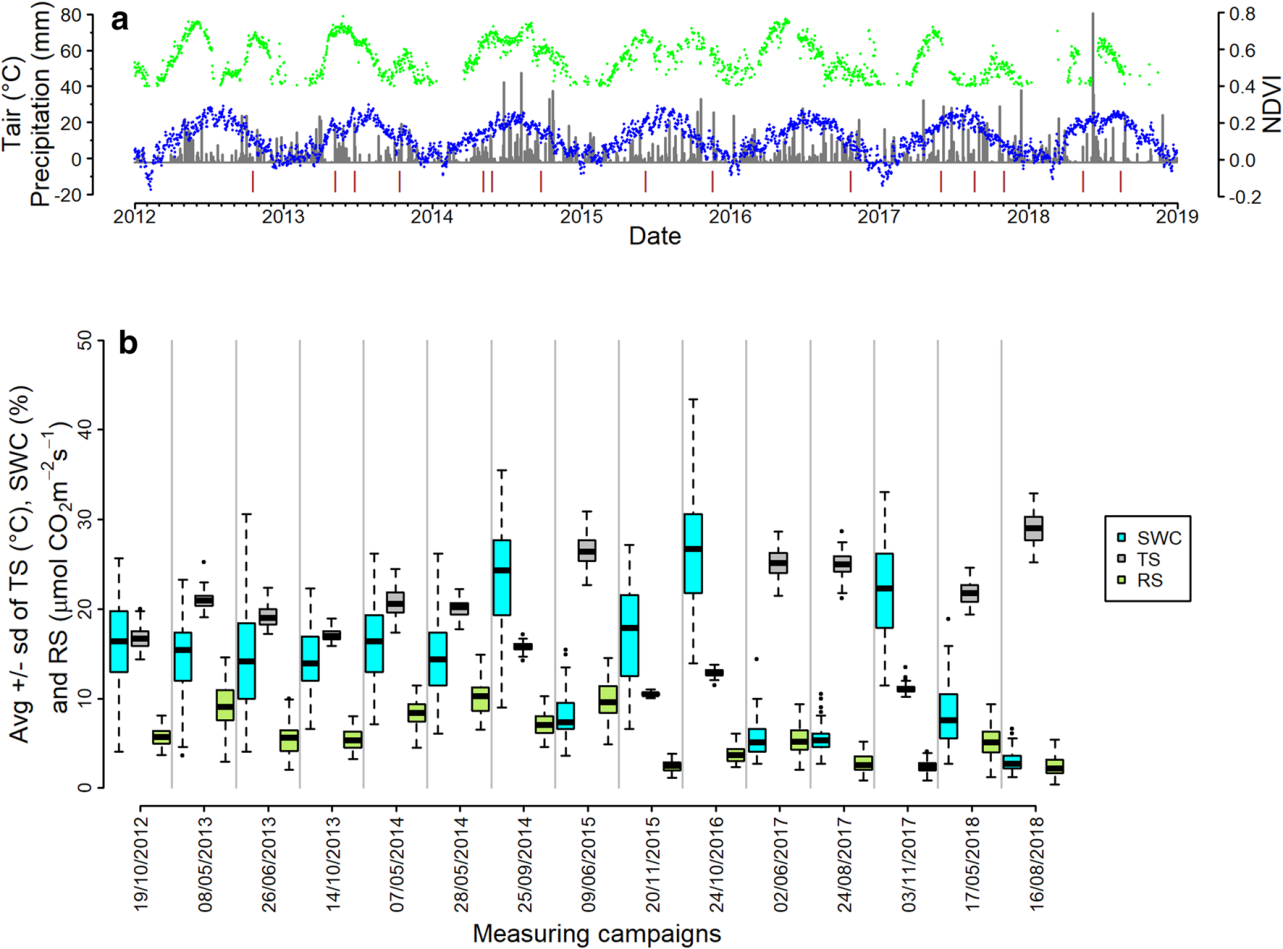
(**a**) Course of daily average temperature (blue), daily precipitation sum (grey) and broad-band NDVI (green) along the study years. Brown sticks show the spatial measuring occasions. (**b**) Boxplot groups of SWC (blue), T_s_ (grey) and R_s_ (green) data along the measuring campaigns.

Daily average temperature, precipitation sum and broad-band normalized difference vegetation index (NDVI)^50^ are presented in Fig. 8a with the measuring campaigns for the entire study period between 2012 and 2018. The first measuring campaign was usually scheduled for the spring-early summer active periods, the second was in the period of summer drought, while the third was in autumn, often along a re-greening period. Several very intensive precipitation events could be distinguished. The actual R_s_ of the measuring campaigns covered wide ranges of the potential activity with different variability from time to time due to a corresponding variability in SWC and T_s_ (Fig. 8b).

### Stability proxies for resistance/resilience interpretation

In order to gain an insight into the stability of the R_s_ patterns we first calculated the average rank of soil respiration (**rankR**_**s**_) for the measuring positions on the basis of the full dataset. This way we could grasp the long-term, persistent distribution of the spatial positions with low or high R_s_ activities. The calculation relied on ranking the measured values from the 1st (the smallest value) to the 78th (the largest value), then averaging the 15 campaigns for each position. Small **rankR**_**s**_ value corresponds to a generally low R_s_ activity in the position, while large **rankR**_**s**_ value means a generally more significant R_s_ activity in that particular position. Another proxy was **rangeR**_**s**_ which we calculated as the difference between the maximum and minimum rank (**rankR**_**s**_ ) for a position, giving low values with more stable patterns, because the R_s_ rank was found to be more constant for the duration of the measuring campaigns, i.e. these positions showed considerable resistance against environmental constraints. Large **rangeR**_**s**_ values referred to more variability in R_s_ activity as well as more resilient functioning.

### DEM processing and terrain attribute calculations

Digital elevation models (DEM) contain bare-earth elevation data in raster grids. Raster data can be processed by different image processing operations, which serve to extract information from the objects, which is a reasonable approach to capture relevant structures when performed at different scales. Pyramid image processing is a multi-scale approach when the image is processed by smoothing and subsampling steps in several runs. We used a specific case of pyramid image processing along the terrain attribute calculations, called the “mixed scaling”^41^, when DEM is smoothed and subsampled and the calculated terrain attributes are upscaled. This method was found to result in fewer artifacts and more straightforward patterns than other techniques^41^.

In our study, we implemented^56^ the method on a 0.2 by 0.2 m input DEM raster (originating from laser scanning) as follows:

1. DEM was progressively smoothed, i.e., aggregated by a factor of two, resulting in six different resolution DEM rasters along a geometric series between 0.2 and 6.4 m (DEM1: 0.2 m, DEM2: 0.4 m, DEM3: 0.8 m, DEM4: 1.6 m, DEM5: 3.2 m, DEM6: 6.4 m), and another scale was also calculated to meet the resolution of the measuring campaigns (10 m, DEM7)
2. Terrain analysis was performed on each of the DEMs, giving firstly a series of terrain attribute rasters with different raster cell sizes/resolutions
3. Terrain attributes were then disaggregated, or upscaled to the original resolution of DEM1, resulting in differently smoothed terrain features with the same raster cell sizes

Six terrain attributes were calculated following the guidelines^35^ for the best characterization of the surface with the least potential co-variance between the selected attributes and the ones which were found to be applicable for a range of terrain complexities:

- local mean elevation (*mALT*),
- standard deviation of elevation (*SD*),
- topographic position index (*TPI*),
- slope (*Sl*),
- Easterness and Northness (*East*, *North*).

Details about the calculations can be found in SI.

### Spatial data processing

In order to visualize stability proxies we performed variography and kriging. The steps of the spatial data processing, detailed description of variography, kriging and leave-one-out cross-validation can be found in the SI (as well as some results, which are only background information for the main focus of the present article). In brief, we performed variogram analysis first^57–62^ on **rankR**_**s**_, **rangeR**_**s**_, **SWC** and **SOC** data. The criterions for variogram model selection were the residual sum of squares (model with maximum SSErr from exponential, Gaussian and spherical), the Nash–Sutcliffe model efficiency coefficient (E > 0.5), and the range of autocorrelation, *a* (the best fit with *a* within the spatial scale of the study site, i.e., *a* < maximum distance of the diagonal of the rectangle that spans the data locations). Two kinds of kriging methods were used for mapping the variables, ordinary kriging (OK), and kriging with external drift (KED). Kriging results were evaluated by means of the leave-one-out cross-validation method^58^, and as the error estimates for OK and KED didn’t show important differences (in terms of cross-validation errors, i.e., normalized root mean squared error (nRMSE), mean error (meanErr) and mean squared deviation ratio (MSDR)) but we lack OK map for **rangeR**_**s**_ because we lack valid variograms, the presented maps are KED maps.

## Supplementary information

Supplementary information 1

Supplementary information 2

Supplementary information 3

Supplementary information 4

Supplementary information 5

## Data Availability

The datasets generated and/or analysed during the current study are publicly available at Figshare repository (10.6084/m9.figshare.12608393).
