# Supplementary figures and images for: Two potential equilibrium states in long-term soil respiration activity of dry grasslands are maintained by local topographic features
Source: Sci Rep. 2020 Aug 31;10:14307. doi: 10.1038/s41598-020-71292-4 (PMC7459112; doi:10.1038/s41598-020-71292-4)

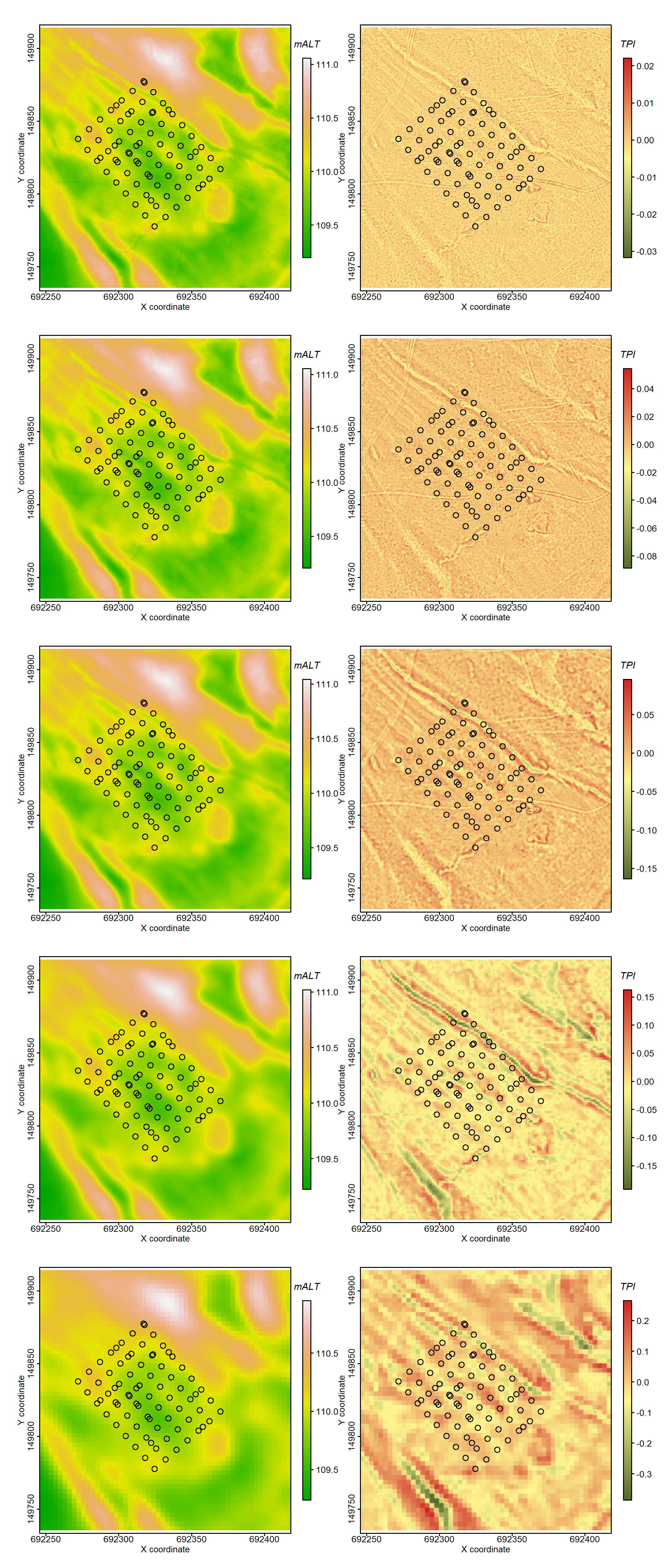

Supplement: Supplementary file 1 — Supplementary information 1 [file 41598_2020_71292_MOESM1_ESM.tiff]

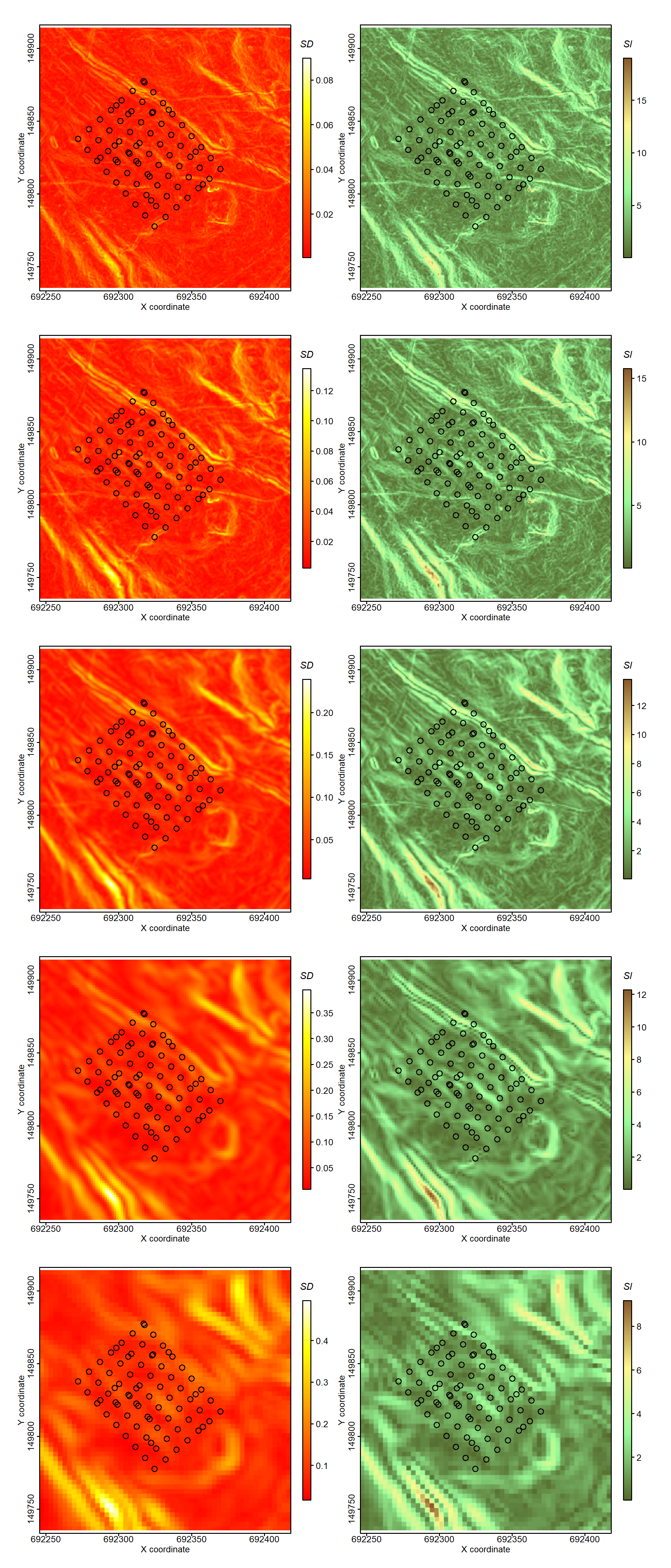

Supplement: Supplementary file 2 — Supplementary information 2 [file 41598_2020_71292_MOESM2_ESM.tiff]

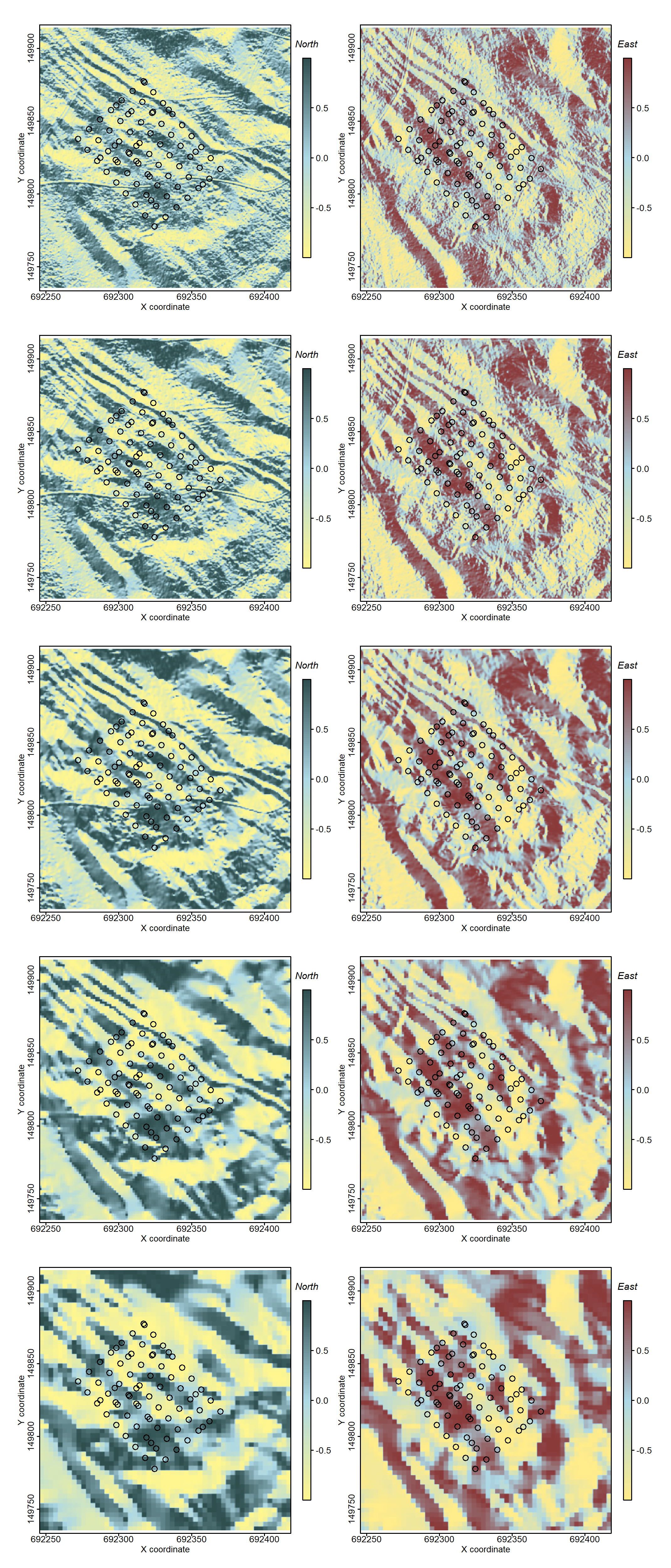

Supplement: Supplementary file 3 — Supplementary information 3 [file 41598_2020_71292_MOESM3_ESM.tiff]
