# Supplementary material for: Two potential equilibrium states in long-term soil respiration activity of dry grasslands are maintained by local topographic features
Source: Sci Rep. 2020 Aug 31;10:14307. doi: 10.1038/s41598-020-71292-4 (PMC7459112; doi:10.1038/s41598-020-71292-4)
Supplement: Supplementary file 4 — Supplementary information 4 [file 41598_2020_71292_MOESM4_ESM.docx]

|  | nrmse | MSDR | meanERR |
| --- | --- | --- | --- |
| rankR_s_ | 0.21 | 1.08 | 0.01 |
| rangeR_s_ | 0.27 | 1.16 | -0.001 |
| meanSOC | 0.12 | 0.85 | -0.02 |
| meanSWC | 0.14 | 1.03 | -0.009 |
