# Supplementary material for: Two potential equilibrium states in long-term soil respiration activity of dry grasslands are maintained by local topographic features
Source: Sci Rep. 2020 Aug 31;10:14307. doi: 10.1038/s41598-020-71292-4 (PMC7459112; doi:10.1038/s41598-020-71292-4)
Supplement: Supplementary file 5 — Supplementary information 5 [file 41598_2020_71292_MOESM5_ESM.pdf]

Two potential equilibrium states in long-term soil respiration activity of dry grasslands are maintained by local topographic features

Szilvia Fóti, János Balogh, Bernadett Gecse, Krisztina Pintér, Marianna Papp, Péter Koncz, Levente Kardos, Mónok Dávid, Zoltán Nagy

Supplementary Information

DEM processing and terrain attribute calculations

The original 0.2 by 0.2 m DEM raster originated from laser scanning, and it was progressively smoothed (cf. DEM1-7 in the manuscript Methods) in a “mixed scaling” [1] approach. In our study, we used 5 × 5 box (equal weights in the filtering matrix) and Gaussian (higher weights for central cells than for peripheral in the filtering matrix) blur kernels as neighbourhood in the terrain attribute calculations for local mean elevation (*mALT*), standard deviation of elevation (*SD*) and topographic position index (*TPI*). For slope (*Sl*) and aspect derived Easterness and Northness (*East*, *North*) we used 8 neighbours, as suggested by Lecours et al. (2017). As we couldn’t find any of the blur kernels superior to the other when considering correlations between raster derived attributes and other variables of our study, the maps will be presented for the box blur kernel calculations for simplicity, and for DEMs 1-5 (cf. correlation analysis in the manuscript).

Local mean elevation

mALT, which is a smoothed elevation data compared to the DEM absolute elevation values, was calculated with the following equation (SI Eq. 1)

$$\bar{z} = \frac{1}{n_R}\sum_{i\in R} z_i, \qquad \text{(SI equation 1)}$$

where $\bar{z}$ is the average elevation within a specific radius (*R*) around a central raster cell from the actually used DEM raster.

Standard deviation of elevation

We also calculated *SD* (SI Eq. 2) of the elevation [3] for the correspondent *R* as follows:

$$SD = \sqrt{\frac{1}{n_R - 1}\sum_{i=1}(z_i - \bar{z})^2}\,, \qquad \text{(SI equation 2)}$$

where z_i values are the elevations of the correspondent *R* radius from the actually used DEM raster cells, while $\bar{z}$ is the mean elevation within *R*. *SD* describes the heterogeneity, local surface roughness [4] within the raster.

TPI

TPI was calculated according to De Reu, J. et al. (2013) as follows (SI Eq. 3):

$$TPI = z_0 - \bar{z}, \qquad \text{(SI equation 3)}$$

where z_0 is the elevation of the central point from the actually used DEM raster, while $\bar{z}$ is the mean elevation within a specific radius (*R*) around that central raster cell as in SI Eq. 1. *TPI* is positive, if the central raster cell is located higher than the surrounding cells within *R*, and is negative, if the central raster cell is located lower than the surrounding cells within *R*. Positive *TPI* and small *SD* means a (local) ridge, while negative *TPI* and little elevation *SD* within *R* means a (local) valley.

Slope

Sl calculates the rate of change in elevation between positions within the corresponding DEM [5], it is the tangent (vertical “rise”/horizontal “run”) of a surface angle to the horizontal in degrees.

Easterness and Northness

Aspect is the compass direction that a slope faces, derived from the maximum downslope rate of change in value from a raster cell to its neighbours. It is a circular variable ranging clockwise from 0° to 360° degrees from due north (both 0° and 360° meaning N facing slope, 90° meaning E facing slope etc. , Ritter, 1987). In ecology, use of sine (Easterness: *East*) and cosine (Northness: *North*) of aspect is more frequent because they provide a continuous gradient of east-west and north-south directions, respectively. Northness and Easterness with the values close to +1 mean that the slope is northward and eastward in general, while values close to -1 mean a generally south- and west-facing slope, respectively.

Spatial data processing

Spatial patterns of average rank of R_s (**rankR_s**), range of ranks by positions (**rangeR_s**), average soil water content (**meanSWC**) and soil organic carbon content (**meanSOC**) were investigated by variograms and then mapped by kriging.

Variography

All variables were standardized to zero mean and unit variance before variography and kriging to facilitate comparison of different variables [6].

Semivariance (γ(h)) was calculated as:

$$\gamma(h)=\frac{1}{2N(h)}\sum_{i=1}^{n}\left[z(s_i)-z(s_i+h)\right]^2 ,\qquad \text{(SI equation 4)}$$

where *z(s)* is a data value at a particular location, *h* is the average separation distance between data pairs, and *N(h)* is the number of data pairs separated at a distance of *h* [7].

Gaussian, exponential and spherical models were fitted to the experimental semivariances against lag distance.

Gaussian:

$$\gamma(h) = y_0 + c\left(1 - e^{\left(\frac{-h^2}{a_0^2}\right)}\right) \qquad \text{(SI equation 5)}$$

Exponential:

$$\gamma(h) = y_0 + c\left(1 - e^{\left(\frac{-h}{a_0}\right)}\right) \qquad \text{(SI equation 6)}$$

Spherical:

$$\gamma(h) = y_0 + c\left[1.5\frac{h}{a_0} - 0.5\left(\frac{h}{a_0}\right)^3\right] \quad ;\text{if } h < a$$

$$\gamma(h) = y_0 + c \quad ;\text{if } h \geq a \qquad \text{(SI equation 7)}$$

In the models, h is the lag distance, a is the autocorrelation length (the distance at which the variogram reaches a plateau or, in the case of models with an asymptotic plateau, at which the variogram reaches its 95%, and is calculated from a_0 as $a=a_0\times3$ in the case of the exponential model, $a=a_0\times3$^0.5 in the case of the Gaussian model, and $a=a_0$ in the case of the spherical model), y_0 is the variance resulting from measurement errors and smaller scale processes ('nugget effect') and c is the structural variance.

The criterion for model selection was the residual sum of squares (SSErr). The goodness of model fit was quantified by the Nash–Sutcliffe model efficiency coefficient (ME), which is calculated similarly to the coefficient of determination, but ranges from $-\infty$, indicating a better prediction of the observed values by the mean than by the model to 1, which points to a perfect match of the observed and modelled data. Only fits with ME≥0.5 were accepted.

Kriging

Kriging is an interpolation technique for the estimation of the values of a variable at unsampled locations, based on the measured values in the neighbourhood and variogram parameters. On the one hand, we used ordinary punctual kriging (OK), in which the kriging neighbourhood was set to the autocorrelation length of the variogram in question [8], and 7 to 25 nearest data [9] within this range were used for the estimation. Variograms with ME≥0.5 values were used which had already been found to be the best fitting models from exponential, Gaussian and spherical. On the other hand, we also run universal kriging or kriging with external drift (KED). This is a technique by which the values of the sparsely measured target variable at unsampled locations are estimated on the basis of a high resolution auxiliary variable. In our study the 0.2 m resolution DEM of the study plot was used as the auxiliary variable. Here, the autocorrelation length of the residual variograms (fitted on the residuals' - received after subtracting the correlation with ALT from the measured variable - semivariances) and the 7 to 25 nearest points were set as kriging neighbourhood. Finally, data were back-transformed to the original scale for mapping.

Kriging results were evaluated using the leave-one-out cross validation [9]. Leave-one-out cross-validation procedure consisted of a series of estimations by omitting the observed data one by one and predicting their values by kriging. The following error estimates were used to compare the different kriging methods' goodness:

- normalized root mean square error (nRMSE):

$$nRMSE = \sqrt{\frac{1}{n}\sum_{i=1}^{n}(y_i - \hat{y}_i)^2}\,/((\max(y) - \min(y))), \qquad \text{(SI equation 8)}$$

where y is the variable in question, y_i is one observed value of the variable at a given position, $\hat{y}_i$ is the estimated value for the given position by the kriging method when the point has been omitted from the dataset, n is the number of observations.

- mean squared deviation ratio (MSDR), because we could compare the goodness of the three methods on the same dataset and choose the method with MSDR closest to 1. This comparison allowed us to assess the importance of the auxiliary variable in the estimation.

$$MSDR = \frac{1}{n}\sum_{i=1}^{n}(y_i - \hat{y}_i)^2/var_{kr},$$ (SI equation 9)

where the parameters are the same as in SI equation 8, and var_{kr} is the kriging variance.

- and with regard to prediction bias: the closer the mean error (meanERR) to zero, the better the prediction is. Its negative values mean over-estimation, whereas its positive values mean under-estimation by the method.

$$MeanERR = \frac{\sum_{i=1}^{n}(y_i - \hat{y}_i)}{n},$$ (SI equation 10)

with the same parameters as SI equation 8.

SI Table 1

Stability proxies, had larger estimation errors than the background factors in terms of nrmse, but quite similar in the other terms. All were adequately good for acceptably mapping the KED estimate of the variables for the entire study plot.

References

1. Behrens, T., Schmidt, K., Macmillan, R. A. & Rossel, R. A. V. Multi-scale digital soil mapping with deep learning. *Sci. Rep.* 2–10 (2018) doi:10.1038/s41598-018-33516-6.
2. Lecours, V., Devillers, R., Simms, A. E., Lucieer, V. L. & Brown, C. J. Towards a framework for terrain attribute selection in environmental studies. *Environ. Model. Softw.* **89**, 19–30 (2017).
3. Alexander Brenning, Bangs, D. & Becker, M. RSAGA: SAGA Geoprocessing and Terrain Analysis. (2018).
4. De Reu, J. *et al.* Application of the topographic position index to heterogeneous landscapes. *Geomorphology* **186**, 39–49 (2013).
5. Ritter, P. A Vector-Based Slope and Aspect Generation Algorithm. *Photogramm. Eng. Remote Sensing* **53**, 1109–1111 (1987).
6. Katsalirou, E., Deng, S., Nofziger, D. L., Gerakis, A. & Fuhlendorf, S. D. Spatial structure of microbial biomass and activity in prairie soil ecosystems. *Eur. J. Soil Biol.* **46**, 181–189 (2010).
7. Dale, M. *Spatial pattern analysis in plant ecology*. *Ecology* (Cambridge University Press, 1999). doi:10.2135/cropsci2001.413916x.
8. Goslee, S. C. Behavior of Vegetation Sampling Methods in the Presence of Spatial Autocorrelation. *Plant Ecol.* **187**, 203–212 (2006).
9. Oliver, M. A. & Webster, R. A tutorial guide to geostatistics: Computing and modelling variograms and kriging. *Catena* **113**, 56–69 (2014).

SI Figure and Table Legends

SI Figure 1: mALT (left hand figure block) and TPI (right hand figure block) along DEMs 1-6 (from top to down) calculated with box blur kernel. Black circles represent the measuring grid.

SI Figures 2-: SD (left hand figure block) and SI (right hand figure block) along DEMs 1-6 (from top to down) calculated with box blur kernel. Black circles represent the measuring grid.

SI Figure 3: North (left hand figure block) and East (right hand figure block) along DEMs 1-5 (from top to down) calculated with box blur kernel. Black circles represent the measuring grid.

SI Table 1: Leave-one-out cross-validation errors for KED estimates of rankR_s, rangeR_s, meanSOC and meanSWC.
